# Exercise and Cognitive Function

**DOI:** 10.3390/jcm8101707

**Published:** 2019-10-16

**Authors:** Paul D. Loprinzi, Ashley Lovorn

**Affiliations:** Department of Health, Exercise Science and Recreation Management, Exercise & Memory Laboratory, University of Mississippi, MS 38677, USA; arlovorn@go.olemiss.edu

Cognitive function is associated with longevity and is of critical importance for optimal daily functioning [1]. As such, the identification of factors that enhance cognitive function is a worthwhile endeavor. To address this important health issue, the present Special Issue in the *Journal of Clinical Medicine* was specifically dedicated to research evaluating the effects of exercise on cognitive function. This Special Issue published 23 articles dedicated to this topic (Table 1). These studies evaluated a variety of cognitive outcomes, including, for example, executive function, episodic memory, emotional memory, motor memory, and false memory. Various biological and structural correlates of cognition were evaluated, such as brain-derived neurotrophic factor (BDNF) and brain gray matter volume. Additionally, the evaluated exercise protocols varied, including, for example, acute exercise bouts, exergaming, comparison of different modalities of exercise (e.g., open- vs. closed-skilled exercise), and chronic exercise training. 

Several interesting findings were observed from this collective body of work. As an example, Chen et al. [2] evaluated the potential dose–response relationship between acute exercise duration and executive function. They observed that a 20 min bout of moderate-intensity treadmill exercise was effective in enhancing aspects of executive function. Other related research has demonstrated that this exercise duration is also associated with improved memory function [3,4,5,6], with the post-exercise recovery period also potentially moderating this effect [7]. Several of the papers published in this Special Issue also provide insights into the underlying mechanisms through which exercise may influence cognition. For example, Hsieh et al. [8] provided evidence that the N450 ERP may be a potential neural correlate through which acute exercise may influence executive function. Relatedly, Winneke et al. [9] demonstrated that moderate-intensity acute exercise sped up neural markers of stimulus evaluation during attentional control processes. Furthermore, in alignment with recent work suggesting that the complexity of the movement pattern may influence cognition [10], in this Special Issue, Hung et al. [11] demonstrated that open-skilled exercise may have a more pronounced effect on neurotrophic production, which may play a critical role in several sub-cognitive parameters, such as memory function [12,13]. Lastly, this Special Issue also published several review papers highlighting recommendations for future research, such as taking into consideration the potential role of biological sex on the exercise–cognition interaction [14], as well as key methodological considerations when utilizing functional near-infrared spectroscopy technology on this topic [15].

## Figures and Tables

**Table 1:** Summary of published papers in this Special Issue.

Reference	Purpose	Study Design	Exercise Protocol	Cognition	Main Results
[2]	To discover the relationship of dose–response and exercise duration and task-switching in older adults.	Counter-balanced design with four sessions	One control visit, three exercise visits. Exercise used a cycle ergometer for 10, 20, and 45 min.	Executive function	20 min bout of moderate-intensity exercise was effective in enhancing executive function. Longer durations were not optimal in enhancing executive function, but did not harm cognition.
[16]	To present the current evidence that exercise can affect cognitive functions in children with and without ADHD, and the neurophysiological mechanisms of this action.	Systematic review	N/A	Multiple cognitive outcomes	The preliminary findings illustrate that exercise can improve cognitive performance in children with presented ADHD, even without a diagnosis. Further studies need to be conducted regarding the parameters of exercise.
[17]	To determine whether there is any influence of dopaminergic pathways on exercise-induced motor memory consolidation.	RCT	2 min warm-up at (50 W and 100 W) on a cycle ergometer. 3 blocks of cycling at 90% or 45% of Wmax, respectively.	Motor memory consolidation	Illustrated that single nucleotide polymorphism (SNPs) have an impact on synaptic dopamine levels and plasticity-regulating proteins in modulating the effect of acute exercise on motor memory consolidation.
[18]	To asses VBM brain changes between two learning conditions in order to better understand the process of neuroplasticity.	Longitudinal study with a factorial and within-group design	During a 1month period, three training sessions occurred per week, with each session lasting 1 h on a 3 meter long slackline.	VBM brain changes	Illustrated that VBM-observed changes in the brain in response to learning the desired task were dependent upon the learning success and the ability to see.
[19]	To assess how effective 10 min of both exercise and meditation was on college students’ cognitive function.	RCT	Exercise was set at a self-selected brisk walk for 10 min on a treadmill.	Executive function	Concluded that there is not sufficient evidence to conclude whether or not acute exercise and meditation can affect cognitive function.
[20]	To examine the effects of acute exercise on reducing proactive memory interference.	RCT	Study 1: 15 min self-selected brisk walk on the treadmill. Study 2: 15 min self-selected jog on the treadmill.	Proactive memory interference	Illustrated that the participants who exercised before memory encoding had the highest number of words recalled; however, this finding was not statistically significant. This trend was only seen after the jogging protocol.
[15]	To summarize the available studies about and methodologies of fNIRS applications.	Systematic, methodology-focused review	N/A	fNIR processing	Illustrated that tools such as fNIRS are pivotal for understanding how various physical activity mechanisms affect cognitive performance. Methodological issues were discussed.
[8]	To explore the possible neural markers that could improve cognitive inhibition after acute exercise.	Counterbalanced, crossover design	5 min warm-up, 20 min moderate intensity aerobic exercise at 60–70% HRR, 5- min cool down.	Inhibition	Suggested that enhanced conflict monitoring via N450 changes could be an underlying process that led to the improved performance in the Stroop test following acute exercise.
[11]	Compared open-skill versus closed skill exercise on BDNF production and task-switching performance.	Counterbalanced design with two testing sessions	5 min warm-up, 30 min of running or badminton in counterbalanced order at a moderate intensity (HRR = 60%)	BDNF and task-switching performance	Illustrated that there is a small moderating role regarding type of exercise and cognitive function. Open-skill exercise produced greater BDNF levels, which correlated with previous studies.
[21]	To investigate aerobic capacity and its associations with cognitive domains of information processing, learning and memory, and verbal fluency	Cross-sectional design	5 min warm-up at 40 W on a bicycle ergometer, followed by an increase in increments of 10 W/min until a self- chosen cadence between 55 to 95 W/min was reached.	Information processing, learning and memory, and verbal fluency	Illustrated limited support for the association between aerobic capacity and most cognitive domains.
[22]	To illustrate the fact that patients suffering from PAD show cognitive dysfunction, and, through exercise, brain function could be protected.	Systematic review	N/A	Cognitive function in patients with PAD	The data supported the idea that it is possible that physical exercise, through various mechanisms, such as myokine secretion and microglial anti-inflammatory phenotype enhancement, could lead to cognition protection in clinical settings.
[14]	To discuss the importance of the sex-specific effects of exercise on memory function	Systematic review	N/A	Memory function	This paper highlighted the potential sex specific differences in memory function and exercise. This led to the discussion of further research to evaluate whether or not sex moderates the effects of acute exercise on memory.
[23]	To compare the effects of coordinative and aerobic training on behavioral and neurophysiological measures of inhibitory control	RCT	Physical education lessons (3 × 45 min) per week, along with a training program (3 × 45 min) per week.	Inhibitory control	Illustrated that there are no concrete differences in the efficiency of aerobic and coordinative training on the enhancement of inhibitory control.
[24]	The goal of this study was to assess the association between global cognitive function, physical function, and non-Aß -dependent factors associated with MCI or AD.	Counterbalanced design with three testing sessions	Assessment of VO_2max_ and a 6 min walking test (6 MWT).	Global cognitive and physical function	Illustrated that global cognitive function and physical function were most closely related to the 6 MWT.
[25]	To assess the potential time effects of acute exercise on true and false episodic memory.	Counterbalanced, randomized, controlled, within-subject design	20 min of exercise on a treadmill with a 5 min recovery period. Minimum speed was 3.0 mph.	False episodic memory	The findings provided evidence that acute exercise, both during and after encoding, may reduce false memories. However, these findings were not statistically significant.
[26]	To investigate how mental fatigue can affect cognitive and aerobic performance in endurance athletes.	Randomized counterbalanced, cross-over design	5–7 min warm-up; MSFT task	Cognitive and aerobic performance	Demonstrated that mental fatigue impairs cognitive function and whole body performance in male endurance athletes.
[27]	To assess the potential effects of sex moderation on exercise-related changes in BMI, cognitive function, and aerobic capacity.	Randomized, parallel-group, observer masked, community-based clinical trials	First 2 weeks: attended YMCA classes 3× per week (could not include aerobic exercise). 24 weeks: 4× per week, 10–15 min of warm-up/cool down and 30–40 min workout (dependent on group) 1. Strength/toning: trainer guided series of core exercise; stretch held for 15–30 seconds. 2. Aerobic: self-selected treadmill, elliptical trainer, stair stepper Week 1–2: 55–65% HRR Week 3–4: 65–75% HRR Week 5–24: 80% HRR	Cognitive function	Illustrated that there was no evidence of sex moderation of aerobic exercise on aerobic capacity or BMI. However, the study showed that for the cognitive outcomes, men benefitted more from aerobic exercise than women.
[28]	To present a complete summary of the various underlying neurophysiological processes of exergame training in older adults.	Systematic review	N/A	Several cognitive outcomes	The review found an overall small but positive influence of exergaming on brain function and cognition in older adults.
[29]	To develop support for a hypothesis that resistance training with BFR could boost the effectiveness of resistance training on cognitive performance.	Hypothesis	N/A	Cognitive function	Concluded that the hypothesis needs to be tested on both short- and long-term parameters in order for concrete evidence and statements to be provided.
[30]	To assess whether or not acute exercise is associated with enhancing emotional memories.	Two-arm, parallel-group (between subject) randomized control design	15 min of a self-selected “brisk- walk” on a treadmill. Minimum speed was set at 3.0 mph.	Long-term emotional memory	Findings illustrated that there is no correlation between acute aerobic exercise and the stimulation of enhanced emotional memory recognition.
[31]	To duplicate and extend the previous research conducted on cognitive benefits for MCI.	Quasi-experimental, within-subject pilot clinical trial	Neuro-exergame	Executive function	Illustrated that an iPad-based neuro-exergame protocol is feasible for MCI to use at home.
[9]	To investigate the immediate effects of acute, moderate-intensity exercise on attentional control.	Crossover design with three testing sessions	20 min of acute exercise, on a stationary bike at 60% of the participants’ VO_2max_.	Attentional control	Illustrated an enhancement effect of acute, moderate-intensity exercise on neurocognitive processes.
[32]	To evaluate whether acute moderate-intensity exercise has any experimental effects on iconic memory or short-term/long-term episodic memory.	RCT	15 min of moderate-intensity exercise on a treadmill at a self-selected “brisk walk” pace.	Iconic memory, short-term episodic and long-term episodic	Illustrated some suggestive evidence hinting that acute aerobic exercise may benefit iconic memory; however, more studies need to be conducted in order to confirm/deny these findings.

AD, Alzheimer’s disease; ADHD, attention-deficit hyperactivity disorder; BDNF, brain-derived neurotrophic factor; BFR, blood flow restriction; BMI, body mass index; fNIR, functional near-infrared; HRR, heart rate reserve; MCI, mild cognitive impairment; MSFT, multistage fitness test; PAD, peripheral arterial disease; RCT, randomized controlled trial; VBM, voxel-based morphometry; YMCA, Young Mens’ Christian Association.
